# Clinical and radiological outcomes of n-HA/PA66 cages in anterior spine reconstruction following total *en bloc* spondylectomy for tumors

**DOI:** 10.3389/fsurg.2023.1278301

**Published:** 2023-12-14

**Authors:** Yuanrui Luo, Peng Xiu, Hua Chen, Jiancheng Zeng, Yueming Song, Tao Li

**Affiliations:** Department of Orthopedics, Orthopedic Research Institute, West China Hospital, Sichuan University, Chengdu, China

**Keywords:** cage, spinal tumor, reconstruction, *en bloc* spondylectomy, surgery

## Abstract

**Objective:**

This retrospective monocentric study was conducted to evaluate the clinical and radiological outcomes of the nano-hydroxyapatite/polyamide66 (n-HA/PA66) cage in reconstructing the anterior column of the spine following total *en bloc* spondylectomy (TES).

**Methods:**

A cohort of 24 patients, 20 diagnosed with primary malignant tumors and 4 with metastatic malignancies, was selected based on specific inclusion criteria. All were subjected to TES and anterior column reconstruction with the n-HA/PA66 cage from January 2013 to July 2023 at a single institution. Pre-operative embolization was performed on all patients. Documented factors included operation duration, intraoperative blood loss, length of hospital stay, treatment history, and involved level. Mechanical complications and radiological parameters such as the local kyphotic angle (LKA), anterior vertebral height (AVH), posterior vertebral height (PVH), cage subsidence, and bone fusion time were evaluated. Quality of life and neurological function were gauged using tools like the Visual Analog Scale (VAS), Eastern Cooperative Oncology Group (ECOG) performance score, Karnofsky Performance Score (KPS) scale, and American Spinal Injury Association (ASIA) grading.

**Results:**

All patients were followed up for 12–127 months, with an average period of 39.71 months. An average operation time of approximately 8.57 h and a blood loss volume of about 1,384 ml were recorded. No instances of tumor recurrence or multiple organ metastases were reported, though recurrence was detected in 2 living patients. Solid fusion was achieved in all patients at a mean time of 6.76 ± 0.69 months. Cage breakage or migration was not observed. Subsidence into the adjacent vertebral bodies was identified in 3 patients but was deemed clinically irrelevant. Significant improvements in VAS, ECOG performance score, KPS scale, and ASIA scores were noted from pre- to post-surgery (*P* < 0.05). A marked enhancement in the AVH was observed from before surgery to immediately after (*P* < 0.05). LKA, AVH, and PVH values between postoperative and final follow-up showed no significant variance (*P* > 0.05).

**Conclusion:**

The integration of TES and the n-HA/PA66 cage was found to yield promising clinical and radiological outcomes in anterior column spine reconstruction. The use of this material did not hinder oncological care, including the provision of adjuvant treatments (chemo/radiotherapy), ultimately contributing to the enhanced long-term quality of life for spinal tumor patients.

## Introduction

Spinal tumors, whether primarily malignant or metastatic, can significantly impair vertebral stability and can even impact nerve roots or the spinal cord, leading to high disability rates. A surgical approach known as TES was introduced by Tomita et al. in 1994 to tackle such conditions (1). This technique, anchored in radical oncological concepts, reduces local recurrence and lengthens survival by compartment-orientated resections of the spine, thus minimizing tumor cell contamination in the surrounding tissues (2, 3). This is then followed by the reconstruction of the anterior column and firm fixation of the posterior column (1). Initially, radical resections were restricted to primary spinal tumors, such as vertebral sarcomas, chondrosarcomas, or chordomas (4–6). The technique was later broadened to include solitary metastases of biologically favorable tumors (1, 7–9). As our understanding of spinal tumors has evolved, and with the advent of innovative treatments and surgical techniques, patients’ overall survival rate has significantly improved (10–12). Consequently, it's essential to consider other clinical aspects like fusion, neurological function, and pain management to enhance patients’ quality of life (13). Post-TES anterior column reconstruction remains a challenging task, especially when tumors extend to paraspinal muscles, ribs, and other surrounding structures that need removal, which can destabilize the spine further (14). It then becomes challenging for spinal surgeons to assess the lifespan of spinal reconstruction. Numerous anterior column reconstruction techniques have been described, such as bone grafts (15, 16), titanium mesh cages (TMC) (17, 18), carbon fiber stackable cages (19), three-dimensional printing porous prosthesis (17), or expandable cages (20). However, definitive evidence about the superiority of one technique over another is lacking (21).

One proposed solution is the nanohydroxyapatite/polyamide 66 (n-HA/PA66) cage, a product composed of nano-scale hydroxyapatite and polyamides. With characteristics resembling human bone, this cage boasts excellent biocompatibility and bone conductivity. It's said to promote bone ingrowth and offers satisfactory bone fusion and spinal stability without escalating the risk of recurrence or surgical complications. However, few studies, mostly with short-term follow-up, have reported on the use of the n-HA/PA66 cage, leaving a gap in the clinical evidence supporting its efficacy in spinal tumor surgery (22). The focus of this study is to determine whether the n-HA/PA66 cage provides an effective and stable solution for anterior spinal column reconstruction following TES in spinal tumor patients.

## Materials and methods

### Patient selection

We conducted a retrospective observational study in accordance with the Declaration of Helsinki and under the local Institutional Review Board's approval (No. 2019–654). All patients provided informed consent. This study involved 24 non-consecutive patients diagnosed with spinal tumors who underwent TES using the n-HA/PA66 cage technique in our institution.

Patients were included based on the following criteria: (1) confirmed diagnosis of a solitary primary spinal tumor or metastatic tumor through various methods, including preoperative anteroposterior and lateral radiographs, computed tomography (CT), magnetic resonance imaging (MRI), positron emission tomography-computed tomography (PET-CT), pathologically preoperative CT-guided biopsy, and postoperative histopathology; (2) tumors that satisfied criteria for Tomita’s classification types I–V (23); (3) patients with a revised Tokuhashi score predicting a survival time of more than six months (24); (4) TES of the involved vertebrae with the anterior spinal column reconstruction using n-HA/PA66 cage.

The exclusion criterion included piecemeal excision of the spinal tumor and metastatic spinal tumor accompanied by systemic metastasis of significant organs.

### Patient evaluation

We evaluated patients based on their clinical information: age, sex, symptoms, neurological findings, preoperative radiographs, CT, MRI, pathological diagnosis, tumor location, and so on. The prognosis was assessed using the revised Tokuhashi score, while the therapeutic choice was determined using Tomita's score. Preoperatively, all patients were evaluated using the Weinstein–Boriani–Biagini surgical staging system (25). We collected data on the operative time, intraoperative blood loss, and blood transfusion volume from the anaesthesia notes.

### Operative procedures

All patients underwent preoperative segmental arterial embolization to minimize intraoperative blood loss.

The surgical approach and technique, dictated by tumor characteristics such as location and size, and its impact on surrounding neurovascular structures, were informed by radiographic studies. Consultations with approach-related surgeons significantly guided these decisions. After the surgical plan received approval, patients were thoroughly informed about the process and potential risks. If preoperative radiotherapy was necessary based on tumor histology, surgery proceeded within a window of 30–40 days post-radiotherapy. Following the administration of general anesthesia, patients were placed in a prone position. The pathological level was identified via an intraoperative radiograph, leading to a dorsal vertical midline incision. The paraspinal muscles were then dissected and drawn back to reveal the affected vertebra and its associated structures. For thoracic vertebra involvement, the corresponding ribs, lateral nerve roots, intercostal nerves, and blood vessels were exposed and isolated to access the pedicle. The process continued with the implantation of pedicle screws, positioned two to three levels above and below the affected vertebra. Position accuracy was confirmed through intraoperative radiographs. Then, all posterior spinal elements were removed in a single unit with the help of ultrasonic bone curette and rongeur. To reduce bleeding and potential tumor cell contamination, the exposed surface was treated with bone wax and the operative area was rinsed with distilled water. Next, a blunt separation of the pleura or retroperitoneal and nearby soft tissues occurred, extending the separation to the discs superior and inferior to the impacted vertebra. The compromised vertebra was carefully removed with a thread-wire saw, followed by discectomies above and below until the bony endplates were revealed. When the lumbar vertebra was involved or the tumor extensively affected the anterior column of the vertebral body and surrounding soft tissues, a combined posterior-anterior approach was adopted. This approach safeguarded the nerve roots at the removal level(s), allowing them to be meticulously separated from the tumor vertebra, along with the adjacent nerve roots, ensuring complete tumor removal. After this, a suitably-sized n-HA/PA66 cage, packed with morselized autologous iliac or rib bone, was selected and inserted. Intraoperative radiography confirmed the correct implantation of the n-HA/PA66 cage and posterior instruments. After one final rinse of the operative field with distilled water and drain placement, the wound was sutured in layers. The removed vertebra was sent for further pathological investigation.

### Postoperative management

Following surgery, spinal radiographs were examined to assess the state of internal fixation. For the first three days, antibiotics were administered as a preventive measure. Patients’ VAS and ASIA grades were evaluated on the third postoperative day. Patients commenced walking 4–6 days post-surgery with recommended rehabilitation for the initial three months. An orthosis was employed for a minimum of three months to ensure complete bone fusion. Adjuvant therapies were performed as needed based on individual pathology. For patients with primary spinal tumors, radiation and chemotherapy or targeted treatment should be initiated 1 month post-surgery. For those with spinal oligometastasis, continue the anti-primary cancer radiation, chemotherapy, or targeted treatment regimen 1 month after surgery, supplemented with bone protectants (bisphosphonates or denosumab) as appropriate.

### Clinical evaluation

Pre-surgery assessments and the last follow-ups were conducted by doctors using the ECOG performance score and the KPS to gauge health-related quality of life and performance status. The neurological function recovery and pain improvement levels of patients should be assessed using ASIA and VAS at preoperative, postoperative, every three months in the first year after surgery, and subsequently every six months, as well as at the final follow-up. Details of complications such as spinal cord and dura injuries, postoperative complications including infection, and instrumentation failure were recorded.

### Radiographical assessment

As part of the oncology follow-up and for the purpose of monitoring implant position and bony fusion, radiographs, and CT or MRI scans were scheduled quarterly for the first year, then semi-annually. Using Cobb's method, the LKA was measured. The vertebral height of the anterior and posterior margins of the diseased vertebral body was also recorded as defined by Lee et al. (26). These assessments, along with bone graft fusion based on the radiologic criteria of Bridwell et al. (15), were performed before and after surgery.

The n-HA/PA66 cage was closely monitored for mechanical complications like migration, subsidence into the adjacent vertebral bodies, or breakage. All measurements were taken by a single independent observer (spine surgeon) not involved in these patients’ surgeries or cases.

### Statistical analysis

Analyses were conducted using SPSS 26.0 software, and all data were expressed as means ± standard deviations (SDs) for parametric analyses. Changes in clinical data before and after surgery were compared using paired *t*-tests. Repeated measures analysis was used for normally distributed data at different times, and the Kruskal–Wallis test was used for non-normally distributed data. A value of *P* < 0.05 indicated statistical significance.

## Results

The patient demographic consisted of an average age of 42.83 ± 13.68 years with 12 males. The average body mass index (BMI) was 22.04 ± 2.37 Kg/m^2^, ranging from 17.15 to 26.30 Kg/m^2^. Among the patients, 20 had primary spinal tumors, and 4 had metastatic tumors. The most common tumor type was the giant cell tumor, followed by osteosarcoma. The Tomita system categorized tumors as 1 tumor of type 2, 4 tumors of type 3, 6 tumors of type 4, and 13 tumors of type 5. There were 12 thoracic tumors, 7 lumbar tumors, and 5 spanning the thoracolumbar junction. All patients underwent single-level TES and were followed up post-surgery.

The patient’s clinical data, tumor characteristics, and treatment information are summarized in Tables 1, 2.

**Table 1:** Clinical data and tumor characteristics in 24 patients.

Patient	Sex	Age (years)	Primary tumors	Metastatic tumors	Tumor location	Preoperative symptoms	Revised Tokuhashi score	WBB staging	Tomita's classification
1	F	50	Bone giant cell tumor	/	T12	Back pain	/	2–6, ABC	Ⅲ
2	M	37	Osteosarcoma	/	T12	Back pain	/	2–11, ABCD	V
3	M	55	/	Renal clear cell carcinoma	L2	Back pain	12	8–11, ABCD	Ⅲ
4	F	45	/	Vulvar epithelioid sarcoma	T6	Back pain	11	1–8,12, ABCD	V
5	F	64	/	Intrahepatic cholangiocarcinoma	T1	Back pain	11	6–9, ABC	V
6	M	28	Chondrosarcoma	/	L5	Back pain	/	3–10, ABCD	V
7	M	57	Chordoma	/	T10	Bilateral leg numbness and fatigue	/	5–8, BCD	V
8	M	38	Langerhans cell histiocytosis	/	T1	Back pain	/	1–2,5–12, ABCD	Ⅲ
9	M	41	Chondrosarcoma	/	T7	Back pain	/	1–2,8–12, ABCD	Ⅳ
10	M	48	Angiomatoid fibrous histiocytoma	/	L2	Back pain	/	4–9, BCD	Ⅳ
11	F	63	Undifferentiated pleomorphic sarcoma	/	L3	Back pain	/	5–8, BC	Ⅲ
12	F	38	Diffuse large B cell lymphoma	/	T11	Back pain	/	3–10, ABCD	V
13	F	39	Solitary fibrous tumor	/	L3	Back pain	/	3–6, BC	Ⅱ
14	M	32	Bone giant cell tumor	/	T11	Back pain + bilateral leg fatigue	/	1–11, ABCD	V
15	M	55	Chordoma	/	T1	Back pain + bilateral leg fatigue	/	4–9, ABCD	V
16	F	53	Chondrosarcoma	/	T9	Back pain	/	7–9, ABC	V
17	M	46	Bone giant cell tumor	/	T6	Back pain	/	3–10, ABCD	V
18	F	46	Osteosarcoma	/	L3	Back pain	/	3–9, BCD	Ⅳ
19	F	28	Bone giant cell tumor	/	T9	Back pain	/	3–10, ABCD	V
20	M	43	/	Left femoral osteosarcoma	T11	Bilateral leg numbness and fatigue	9	1–3,7–11, BCD	Ⅳ
21	M	63	Leiomyosarcoma	/	T10	Back pain	/	3–9, ABCD	V
22	F	21	Tenosynovial giant cell tumor tumor	/	T6	Back pain + bilateral leg fatigue	/	1,7–12, BCD	Ⅳ
23	F	25	Bone giant cell tumor	/	L5	Back pain + bilateral leg numbness	/	4–9, BCD	Ⅳ
24	F	13	Fibrosarcoma	/	T8	Back pain	/	1–9,12, ABC	V

F, female; M, male; WBB, Weinstein–Boriani–Biagini.

**Table 2:** Treatment information of 24 patients.

Patient	Sex	Age (years)	Previous treatment	Postoperative treatment	Surgery approach	Tumor recurrence	Oncological status	Cage subsidence
1	F	50	None	Denosumab	P	No	CDF	/
2	M	37	None	RT + CHT	P	Yes	AWD	Lower-end plate: 4 mm Upper-end plate: 3 mm
3	M	55	Surgery for primary tumor+TT	TT + Denosumab	P + A	No	CDF	Lower-end plate: 4.5 mm
4	F	45	Surgery for primary tumor+CHT	CHT	P	No	CDF	/
5	F	64	Surgery for primary tumor+CHT	RT + CHT	P + A	No	CDF	/
6	M	28	None	RT	P + A	No	CDF	/
7	M	57	None	RT	P + A	No	CDF	/
8	M	38	None	/	P + A	No	CDF	/
9	M	41	None	/	P	No	CDF	/
10	M	48	None	RT	P + A	No	CDF	/
11	F	63	None	/	P + A	No	CDF	/
12	F	38	None	RT + CHT	P	No	CDF	/
13	F	39	None	RT	P + A	No	CDF	Lower end plate: 3.2 mm
14	M	32	None	Denosumab	P	No	CDF	/
15	M	55	None	RT + Zoledronic acid	P + A	No	CDF	/
16	F	53	None	/	P	No	CDF	/
17	M	46	None	Denosumab	P	No	CDF	/
18	F	46	None	RT	P + A	No	CDF	/
19	F	28	None	Denosumab	P + A	No	CDF	/
20	M	43	Surgery for primary tumor	RT	P	No	CDF	/
21	M	63	None	RT + TT	P + A	Yes	AWD	/
22	F	21	None	RT	P	No	CDF	/
23	F	25	None	Denosumab	P + A	No	CDF	/
24	F	13	None	/	P	No	CDF	/

F, female; M, male; RT, radiation therapy; CHT, chemotherapy; TT, targeted therapy; CDF, continues disease free; AWD, alive with disease; P, posterior approach; A, anterior approach.

### Surgical results

The average surgery duration was around 8.57 ± 2.10 h, with a range of 5.00–15.30 h. Blood loss averaged 1,384.48 ± 794.75 ml, within a range of 400–3,500 ml. The mean transfusion of red cell suspension and fresh plasma was 4.9 ± 3.2 units and 367.24 ± 343.11 ml, respectively. Fixed segments averaged five per surgery. Patients stayed in the intensive care unit for an average of 1.24 ± 1.21 days, and the average postoperative hospital stay was 11.14 ± 3.15 days.

### Complications

Dural tears with cerebrospinal fluid leakage occurred in 3 patients; the tears were covered intraoperatively by fascial tissue, and a drainage tube was placed and removed after at least 7 days. One patient experienced a rupture of the lumbar segment vein under the armpit, partially involving the inferior vena cava, the tear was covered intraoperatively with the assistance of a vascular surgeon. None of the patients had an infection of the surgical wound but one patient developed wound fat liquefaction. Following an intensified dressing change regimen for 6 days, the condition significantly improved, leading to satisfactory wound healing. Additionally, three patients experienced pneumonia accompanied by hydrothorax after surgery; all of these cases improved following antibiotic symptomatic treatment, thoracic puncture drainage, and systematic respiratory function training.

### Clinical results

Neurological outcomes of all patients have improved after surgery apart from 7 cases, who were rated as preoperation at the final follow-up (Table 3). The VAS score decreased from 6.00 ± 0.65 preoperatively to 4.34 ± 0.48 at 3 days after operation, and to 2.72 ± 0.45 at the final follow-up (*P* < 0.001). The KPS increased from 41.40 ± 2.59 preoperatively to 81.03 ± 9.76 at final follow-up, (*P* < 0.001). The ECOG performance score decreased from 1.21 ± 0.49 preoperatively to 0.76 ± 0.51 at final follow-up, (*P* < 0.001). No patients succumbed to tumor recurrence or multi-organ metastasis, yet recurrence was observed in two individuals. Both patients, farmers from economically disadvantaged regions, could not adhere to post-operative rest or the systematic treatment advised by bone oncologists due to financial constraints. Presently, they are undergoing radiotherapy and chemotherapy for tumor recurrence.

**Table 3:** Surgical results.

Operating duration (hour)	8.57 ± 2.10
Blood loss (ml)	1,384.48 ± 794.75
Transfusion of blood	
Red cell suspension (unit)	4.9 ± 3.2
Fresh plasma (ml)	367.24 ± 343.11
Average fixed segment (*n*)	5
Stay in the tensive care unit (days)	1.24 ± 1.21
Postoperative hospital stay was (days)	11.14 ± 3.15
Complication (*n*)	
Dural tear	3
Wound fat liquefaction	1
Pneumonia with hydrothorax	3
Vein rupture	1
Follow-up (months)	39.71 ± 35.61

**Figure 1:**
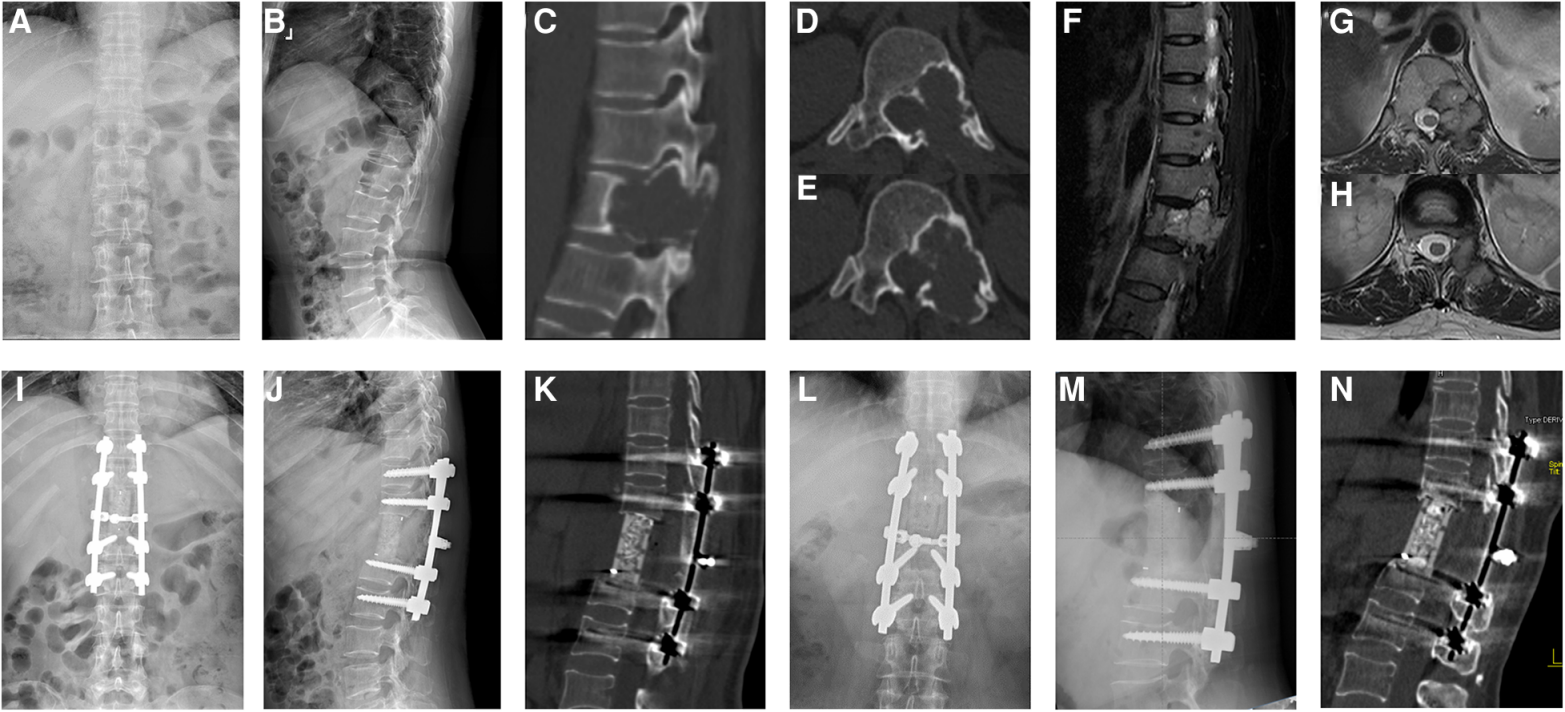
A 50-year-old female patient with a T12 giant cell tumor complicated by neurological deficits. The preoperative anteroposterior (**A**) and sagittal x-ray (**B**), sagittal (**C**) and axial CT (**D,E**), and sagittal (**F**) and axial MRI (**G,H**) scans showed pathological bone destruction of the T12 vertebral body with left intervertebral foramen invasion. The immediately postoperative plain radiographs (**I,J**) and sagittal CT scan (**K**) showed corpectomy, screw fusion, and stable spinal alignment. The plain radiographs (**L,M**) and sagittal CT scan (**N**) showed the absence of instrumentation failure, tumor recurrence, and cage subsidence at the final follow-up.

The surgical results, complications, and clinical results are shown in Table 3 and Table 4, and an illustrative case is shown in Figure 1.

**Table 4:** Comparison of the preoperative and postoperative neurological statuses.

	ASIA grading				
	A	B	C	D	E
Preoperation	0	0	7	16	1
Final Follow-up	0	0	0	14	10

ASIA, American Spinal Injury Association.

### Radiographic results

In this study, a paramount outcome was the achievement of solid fusion in all patients, evidenced radiologically, with a mean time of 6.76 ± 0.69 months. Another critical observation was cage subsidence: 3 out of 24 (12.5%) patients exhibited cage subsidence into the adjacent vertebral bodies at the distal bone–implant interfaces. One of these patients also experienced subsidence at the proximal bone–implant interfaces. However, all three cases of subsidence were clinically insignificant, being asymptomatic with no detrimental effect on posterior instrumentation. Notably, no cases of cage breakage or migration were reported.

As supplementary findings, postoperative correction was consistently achieved across patients. The LKA showed a shift from 14.44 ± 9.92° preoperatively to 12.98 ± 12.49° postoperatively and 13.19 ± 12.46° at the final follow-up (*P* > 0.05). The AVH transitioned from 3.51 ± 3.34 mm preoperatively to 3.91 ± 3.68 mm postoperatively (*P *<* *0.05), settling at 3.63 ± 3.43 mm during the final evaluation. Similarly, the PVH evolved from 3.61 ± 3.63 mm before the surgery to 3.71 ± 3.80 mm after the procedure and finally to 3.67 ± 3.73 mm at the last follow-up (*P* > 0.05). Throughout the course, no discernible loss in height correction or increase in kyphosis was observed, given the consistent parameters between postoperative assessments and the final follow-up.

The postoperative radiological data are shown in Table 5.

**Table 5:** The postoperative radiological data of 24 patients .

	PRE	POST	LFU
AVH	3.17 ± 1.58	3.66 ± 1.46*	3.53 ± 1.39
PVH	3.31 ± 1.18	3.46 ± 1.17	3.37 ± 1.10
LKA	13.33 ± 13.66	11.3 ± 9.61	12.23 ± 8.88
Bone fusion time (month)	6.7 ± 0.6		

AVH, anterior vertebral height; PVH, posterior vertebral height; LKA, local kyphotic angle; PRE, preoperation; POST, postoperation; LFU, last follow-up.

* Compared with preoperative (*P *< 0.05).

## Discussion

Over the past few decades, the treatment of spinal tumors has significantly advanced due to scientific-technological progress. Not only has technology improved diagnostic precision and surgical procedures, but advancements in systemic disease treatments have also enhanced both short and long-term prognoses for spinal tumors (27, 28). Yet, due to their complex anatomical structures and the associated risk of injuring surrounding neural structures, spinal tumors still present formidable treatment challenges compared to limb bone tumors. The widely accepted surgical procedure for spinal tumors, total *en bloc* spondylectomy, comes with its own set of challenges (27, 29–31), one of the most prominent being the significant risk of extensive intraoperative bleeding.

Preoperative strategies are crucial in spinal tumor surgeries, especially when it comes to mitigating the risks of intraoperative bleeding. Erythropoietin (EPO) administration prior to surgery can stimulate the bone marrow to produce more red blood cells, thereby elevating the patient's hemoglobin levels. Especially for spinal tumor surgeries, which often come with significant bleeding risks, boosting a patient's hemoglobin levels can potentially decrease the need for transfusions (32–34). However, it's essential to balance the potential benefits with the risks, as EPO can also increase the risk of thromboembolic and cardiac events (35, 36). Preoperative embolization is another pivotal strategy. By occluding the tumor's blood supply, one can significantly reduce intraoperative bleeding. This technique is particularly useful for highly vascularized tumors (36–38). Nevertheless, its success heavily depends on the tumor's vascularity and location, and there might be potential complications, such as non-target embolization, which need to be carefully weighed (39). Lastly, preoperative imaging plays a paramount role. Modern imaging modalities such as MRI and CT angiography provide detailed insights into the tumor's size, location, and relationship with surrounding structures (39, 40). It assists surgeons in surgical planning, understanding potential challenges, and anticipating areas of significant bleeding. Together, a combination of these strategies can significantly mitigate the challenges and risks associated with intraoperative bleeding in spinal tumor surgeries.

Additionally, since this TES procedure causes a complete loss of spinal continuity and exposure of the spinal cord and nerves, solid spinal reconstruction and complete bone union are essential for absolute stability and safety (41–43). Furthermore, potential issues such as implant loosening, hardware failure, and a delayed healing process can arise due to prolonged instability post-surgery (14). Achieving fast and complete bone union may also be complicated by the patient's overall health condition.

Upon successful tumor resection, surgeons confront two major challenges during spinal reconstruction: bone deficit and extreme instability of the spine. Several reconstruction options exist, each with unique features requiring a careful evaluation of their advantages and disadvantages. Autogenous bone grafts, for instance, are cost-effective with excellent osteointegration potential. However, they present difficulties in connecting to posterior instrumentation and require anterior plating for protection against segmental kyphosis during the creeping substitution phase. The limited harvestable bone restricts autograft use (44, 45), while allografts are prone to late graft collapse, graft fracture, and nonunion so implantation of the allograft bone provides less stability (46). Disease transmission is another theoretical concern of allografts (47). In case of tumor recurrence, the allogenic or autogenous bone graft may suffer erosion, possibly leading to anterior and middle column instability, severe kyphosis, or nerve spinal cord damage.

Polymethyl-methacrylate (PMMA), a prevalent reconstruction technique combines mesh cages packed with cancellous bone grafts, which enables immediate weight-bearing and osteointegration and offers good compression resistance and cost-effectiveness. However, theoretical and practical disadvantages of PMMA often include thermal injury, dislodgement, and extravasation (48). The temperature of the exothermic reaction, additionally, can be dangerous as it reaches over 100°C at the bone-cement interface (49, 50). Despite claims of its suitability primarily for patients with a shorter life expectancy due to questionable osteointegration potential, some long-term outcome reports challenge this assumption (51).

Modular carbon fiber stackable cages are commonly used in oncology cases, as their low atomic number minimizes postoperative imaging scatter, facilitating optimal radiation therapy protocols. While they permit excellent fusion, the high cost of implants may limit their use. Another issue is the cage's inability to preserve lordosis. According to Rousseau et al., the rigidity of the posterior instrumentation and the height of the cage were both connected to substantial changes in local lordosis (52).

The last decade has seen the advent of expandable cages, which promise ease of implantation and *in situ* expansion through minimally invasive approaches. However, metallic artifacts might make the radiographic analysis of fusion following expandable cage implantation challenging. The density of implanted bone will decrease with *in situ* expansion, and the defect may be too wide for bone regeneration to occur across the gap, making it doubtful that bony fusion can be accomplished with expandable cages (53).

Currently, the titanium mesh cage is the preferred choice for reconstruction, but fixation failures such as implant subsiding, intervertebral collapse, and hardware loosening are not uncommon (17, 54).

Three-dimensional printing (3DP), also known as additive manufacturing, has emerged as a potential solution. With its capability to print materials layer by layer into a three-dimensional structure, it offers products such as a 3DP vertebral prosthesis made of titanium alloy (Ti6Al4V). This prosthesis boasts good biocompatibility, and stress shielding reduction, and promotes bone ingrowth, thereby providing reliable initial stability and reducing subsidence to some extent after TES (55). However, fixation failures and implant subsidence remain issues, and the cost of the procedure is prohibitively high for many in developing countries (17, 56, 57).

In summary, the primary goals of reconstruction are to restore the anterior column's load-bearing capacity, fill the bone loss, and correct any deformity caused by the disease. The capacity for the prosthesis to integrate with the host bone or osteointegration is crucial for long-term reconstruction success. Recently, artificial composite materials have gained traction in spinal fusion surgeries. An ideal bone graft substitute material should exhibit good biocompatibility, optimal compressive and flexural mechanical strength, strong bone conduction and osteoinductive properties, no interference with imaging radiation, and be low-cost.

The n-HA/PA66 cage is a composite construct, composed of nano-hydroxyapatite (a key component of natural bone) and polyamide66. Nano-hydroxyapatite is first nanocrystallized and then efficiently dispersed into polyamide66, yielding a material with the durability of hydroxyapatite and the flexibility of polyamide66. Numerous studies substantiate the composite's biocompatibility, safety, and ability to facilitate osteoconduction and provide biomechanical stability (58–63). Experimental evidence from animal models indicates that post-implantation, the cage releases Ca2 + and PO43- ions from its surface. These ions then gradually crystallize on the cage's surface, bridging the gap between the graft and the implant bed and serving as a scaffold for osteogenesis (64, 65). The cage's innovative design includes 2 mm holes in the walls and grooves, theoretically allowing the invasion of vessels, growth factors, and osteogenic factors, thereby enhancing bone healing and promoting bony fusion. Though our study did observe cage subsidence into the adjacent vertebral bodies in 3 out of 24 patients, these instances were clinically insignificant and had no impact on the posterior instrumentation. Meanwhile, it's essential to highlight that for patients who were enrolled as recently as a year ago and as far back as about a decade ago, there were no occurrences of n-HA/PA66 cage collapse, splitting, or fractures during the follow-up periods. Their neurological functions, pain relief, and quality of life not only exhibited marked improvements compared to the preoperative phase but also sustained over the long haul. This underscores that the cage's mechanical robustness is apt for postoperative anterior spinal reconstruction and is instrumental in augmenting the comprehensive health of the patients.

In general, in the reconstruction of stability post-total *en bloc* spondylectomy in spinal tumors, the n-HA/PA66 cage offers distinct advantages. It has a design that reduces local pressure, avoiding collapse and subsidence, making it long-term stable of spinal alignment. Moreover, the n-HA/PA66 cage features multiple small holes in the surrounding wall, promoting capillary growth and facilitating bone fusion. And it's biomechanical properties align closely with human cortical bone, reducing subsidence. Our study found no significant loss of vertebral height, local kyphotic angle, or consequential cage damage at the last follow-up. The cage's radiopaque nature allows for easy bone graft fusion observation, and its construction from bioactive materials makes it a cost-effective and patient-friendly alternative to metal implants like titanium mesh.

Despite promising initial results, our study has several limitations, including a small and heterogeneous sample size and variations in bone-forming abilities. While these findings are encouraging, future research should assess long-term outcomes, warrant further investigation, and compare this technique with others. Additionally, there is a need for further exploration of its performance under radiotherapy, especially in comparison to materials such as carbon, to provide a comprehensive understanding of its suitability among patients undergoing radiation treatment.

## Conclusion

The n-HA/PA66 cage's advantages include enhanced bone fusion, ideal spinal stability, and no increased risk of recurrence or complications. The method of combining TES with the n-HA/PA66 cage appears safe, easy, and effective, potentially improving the long-term quality of life in patients with spinal tumors without hindering oncological care.

## Data Availability

The raw data supporting the conclusions of this article will be made available by the authors, without undue reservation.
